# Decoding the microbial blueprint of pancreatic cancer

**DOI:** 10.3389/fmed.2026.1737582

**Published:** 2026-01-23

**Authors:** Jhommara Bautista, Ricardo Bedón-Galarza, Francisco Martínez-Hidalgo, Martina Masache-Cruz, Melanie Benítez-Núñez, Camila Valencia-Arroyo, Andrés López-Cortés

**Affiliations:** 1 Cancer Research Group (CRG), Faculty of Medicine, Universidad de Las Américas, Quito, Ecuador; 2 Servicio de Medicina Interna, Hospital General Docente de Calderón, Quito, Ecuador; 3 Facultad de Ciencias Médicas, Universidad Central del Ecuador, Quito, Ecuador

**Keywords:** *Aggregatibacter actinomycetemcomitans*, enterobacter, fecal microbiota transplantation, *Fusobacterium nucleatum*, immunomic, *Malassezia* spp., metabolomic, metagenomic

## Abstract

Pancreatic cancer (PC) represents one of the most formidable challenges in oncology, characterized by its asymptomatic onset, delayed clinical detection, and dismal prognosis. Among pancreatic neoplasms, pancreatic ductal adenocarcinoma (PDAC) accounts for over 90% of cases and remains the most aggressive form, driven by late diagnosis, intrinsic chemoresistance, and a profoundly immunosuppressive tumor microenvironment. Recent advances have reframed the human microbiome not as a passive bystander but as an active architect of pancreatic tumor biology. This review delineates the mechanistic axes through which microbial ecosystems orchestrate PDAC progression across four key anatomical niches-gastrointestinal, oral, urogenital, and intrapancreatic. We elucidate how microbial dysbiosis fosters oncogenesis through immune evasion, metabolic reprogramming, and chronic inflammation, implicating specific taxa such as *Fusobacterium nucleatum*, *Malassezia* spp., and *Porphyromonas gingivalis* in immune suppression and chemoresistance. Microbial enzymatic inactivation of gemcitabine and modulation of cytokine networks further underscore the microbiome’s pivotal role in therapeutic failure. Conversely, commensal and probiotic species may potentiate immunosurveillance and enhance treatment efficacy. This review also explores microbiota-derived biomarkers for early detection and the translational promise of microbiome-targeted interventions, including fecal microbiota transplantation, probiotics, and selective antibiotics. By decoding the microbial blueprint of PC, we propose a paradigm in which the microbiome emerges as both a biomarker and a therapeutic axis, offering novel avenues for precision oncology. Furthermore, this integrative synthesis emphasizes the multi-omic, immunometabolic, and therapeutic dimensions of the pancreatic cancer-microbiome interface, where metagenomic, transcriptomic, metabolomic, and immunomic layers converge to shape tumor evolution and therapeutic response, advancing the vision of microbiome-informed precision oncology.

## Introduction

As one of the most aggressive human malignancies, PC remains a formidable clinical and biological challenge marked by silent onset, delayed detection, and profound therapeutic resistance. The pancreas is a vital organ that performs both exocrine and endocrine functions, playing a crucial role in digestion, nutrient absorption, and metabolic regulation. However, its deep anatomical location and the asymptomatic nature of early disease stages often delay diagnosis until the malignancy is advanced and incurable (1). Among pancreatic neoplasms, PDAC accounts for over 90% of cases and is one of the most lethal solid tumors. This malignancy is marked by a complex and immunosuppressive TME that promotes metabolic reprogramming and facilitates interactions between diverse cellular populations, contributing to rapid progression and limited therapeutic response (2). Globally, it ranks as the 12th most diagnosed cancer, yet its mortality rate remains disproportionately high (3). Survival rates for PDAC are alarmingly low, with only ∼18% of patients surviving 1 year after diagnosis and just 11% surviving 5 years (4, 5). These outcomes are largely attributed to delayed detection and intrinsic resistance to therapy.

Imaging remains the cornerstone of PC diagnosis. Multidetector computed tomography (CT) is the preferred modality for initial evaluation, while ultrasonography and magnetic resonance imaging (MRI) provide complementary anatomical resolution (6, 7). In efforts to overcome diagnostic delays, innovative tools such as the PAC-MANN blood-based assay have been introduced. This platform utilizes magnetic nanosensors to quantify protease activity, and when combined with CA 19-9, achieves 85% accuracy in detecting early-stage PC using only 8 μL of blood. Delivering results within 45 min, it represents a rapid, cost-effective, and scalable strategy, particularly advantageous for resource-limited settings (8).

Beyond classical diagnostics, the human microbiome has emerged as a fundamental regulator of systemic physiology and oncogenesis. Comprising bacteria, viruses, and fungi, this “hidden organ” orchestrates host metabolism, immunity, and inflammation (9–15). The intestinal microbiome, in particular, is indispensable for lifelong immune development and tolerance. Disruption of its homeostasis, termed dysbiosis, has been implicated in gastrointestinal, metabolic, cardiovascular, and neuropsychiatric disorders (9, 16–18). Microbial ecosystems also inhabit anatomical regions once considered sterile. The genitourinary tract, for instance, hosts a unique microbiome influenced by age, hormonal milieu, and host-specific factors, though reproducibility across studies remains limited due to methodological variability in sampling and sequencing (19). Increasingly, evidence indicates that the gastrointestinal microbiome plays a critical role in pancreatic tumorigenesis. Alterations in microbial composition and function can modulate local and systemic inflammation, immune regulation, and xenobiotic metabolism-mechanisms directly relevant to PC initiation and therapeutic resistance (20–23).

Recent translational frameworks now position the microbiome as a mechanistic and clinically actionable component of oncology, advancing from correlation to intervention-ready models. Multi-omic evidence links microbial pathways, nucleotide salvage, bile-acid remodeling, and tryptophan–kynurenine metabolism to immune evasion, stromal remodeling, and therapeutic resistance in PDAC, supporting the development of microbiota-informed diagnostics and adjunctive strategies such as live biotherapeutics and precision nutrition (24, 25). For low-biomass niches like the intrapancreatic and biliary microbiomes, standardized sampling, contamination control, and functional readouts (metatranscriptomics, metabolomics) are essential for reproducible biomarker discovery (13, 26). Parallel evidence indicates that early-life microbial imprinting, shaped by delivery mode, breastfeeding, and maternal microbiota, programs immune tone and metabolic set-points that persist into adulthood. Early dysbiosis, particularly altered *Bifidobacterium* and *Lactobacillus* transmission, may predispose individuals to chronic inflammation and metabolic reprogramming conducive to PDAC development. Integrating function-first profiling with life-course microbial determinants could refine PDAC risk stratification and accelerate translation from discovery to personalized interventions (27, 28).

Within the urogenital niche, dysbiosis has been linked to urinary pathologies, whereas dominance of *Lactobacillus* species such as *L. crispatus* supports urogenital health, while *L. gasseri* is often associated with dysbiotic states, reflecting species-specific effects on mucosal stability (29). Similarly, the oral microbiome contributes to pancreatic carcinogenesis, with pro-inflammatory taxa including *Porphyromonas gingivalis* and *Aggregatibacter actinomycetemcomitans* potentially translocating to the pancreas via hematogenous or lymphatic routes and altering its immune milieu (30). Furthermore, the intrapancreatic microbiome, enriched with *Fusobacterium*, *Pseudomonas*, *Enterobacter*, and *Malassezia*, has been shown to shape immune cell infiltration, influence chemotherapeutic efficacy, and modulate disease aggressiveness and prognosis (22).

Recent metagenomic profiling has revealed that PDAC harbors a distinct microbial ecosystem with functional links to tumor metabolism and immunosuppression. Multi-omic integration demonstrates that specific microbial taxa contribute to reprogramming of amino-acid and lipid metabolism, while simultaneously dampening interferon signaling and cytotoxic lymphocyte infiltration (31). These findings suggest that microbial metabolic pathways operate as *co-drivers* of pancreatic tumor biology, aligning with emerging evidence that the gut–pancreas axis orchestrates oncogenic inflammation and immune escape (32).

Beyond initiation and immune escape, the microbiome actively engineers metastatic fitness and organotropism in PDAC. Gut and intratumoral taxa calibrate EMT, desmoplasia, angiogenesis, and immune surveillance, conditioning pre-metastatic niches and shaping therapy response. In parallel, tumor-derived extracellular vesicles encode integrin “zip codes” that direct tissue targeting, such as α6β4/α6β1 biasing lung uptake and αvβ5 engaging liver Kupffer cells, remodeling distant sites via cytokine induction, ECM dynamics, vascular leak, and immune suppression. This tumor-microbe-host circuitry also alters drug bioavailability and ICI efficacy: gut metabolites (SCFAs, bile-acid and tryptophan derivatives) reprogram endothelium and myeloid compartments, while *Gammaproteobacteria* can inactivate gemcitabine; circulating microbial DNA/metabolite signatures capture these states and predict outcomes. Operationalizing organotropism, especially PDAC’s liver/peritoneal tropism, by pairing microbiome-informed biomarkers with EV-guided niche biology could sharpen surveillance, risk stratification, and treatment timing (12, 33).

This review synthesizes the mechanistic dimensions through which the microbiome shapes PC biology across four interrelated niches-gastrointestinal, oral, urogenital, and intrapancreatic. We elucidate how microbial communities modulate the pancreatic TME, influence immune signaling and metabolism, and alter treatment responsiveness via drug biotransformation and immune regulation. Collectively, these insights highlight the microbiome’s growing significance as both a diagnostic biomarker and a therapeutic target in the evolving landscape of PC management.

## Gastrointestinal microbiome

**FIGURE 1:**
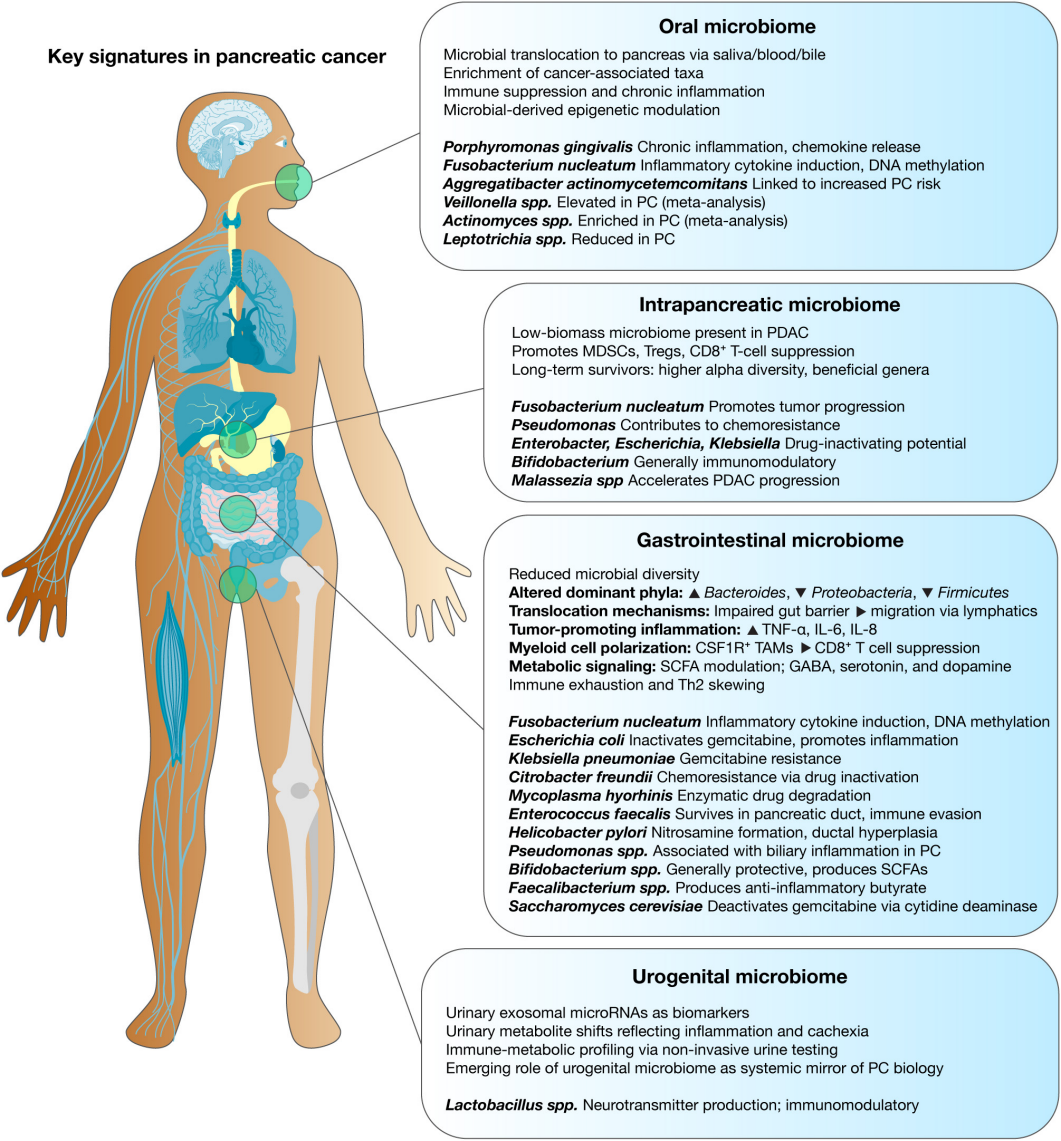
Microbiome-derived signatures across body sites modulating pancreatic cancer. Distinct microbial communities from the oral, urogenital, gastrointestinal, and intrapancreatic niches contribute to the pathogenesis and progression of pancreatic ductal adenocarcinoma (PDAC). Oral microbiota, including *P. gingivalis* and *F. nucleatum*, are associated with chronic inflammation and poor prognosis. Gastrointestinal microbes, such as *H. pylori* and *E. coli*, influence tumorigenesis through immune modulation and genotoxic effects. Urogenital dysbiosis, featuring *Enterococcus faecalis* and *Lactobacillus iners*, may contribute to systemic inflammation and immune escape. The intrapancreatic microbiome, dominated by *Proteobacteria*, *Firmicutes*, and *Malassezia* species, directly shapes the tumor microenvironment by promoting immunosuppression, metabolic rewiring, and chemoresistance.

Among the various microbial ecosystems in the human body, the gastrointestinal microbiome emerges as a critical regulator of systemic immunity, metabolic signaling, and inflammatory pathways (16) (Figure 1). These functions are increasingly implicated in the pathogenesis of PC. Alterations in gut microbial composition, particularly reduced diversity, have been observed in patients with PC. It has been reported a significant shift in gut microbiota populations, characterized by an increased abundance of *Bacteroides* and a decreased proportion of *Proteobacteria*, compared to healthy controls (34, 35). Emerging data link gut microbiota dysbiosis to tumor-promoting inflammation, including enhanced expression of pro-inflammatory cytokines such as tumor necrosis factor-alpha (TNF-α), a hallmark of chronic inflammatory states (36). Although transient inflammation is protective, its persistence fosters a pro-carcinogenic microenvironment that supports malignant transformation.

Systemic and local inflammatory pathways converge in PC, where metabolic disorders such as obesity, type 2 diabetes, and chronic pancreatitis exacerbate gut microbiota dysbiosis and compromise intestinal barrier integrity. This disruption facilitates microbial translocation to pancreatic tissue through mesenteric or lymphatic routes (37). Integrative metabolomic analyses reveal that depletion of *Faecalibacterium* and *Akkermansia* perturbs bile-acid and tryptophan pathways, activating pro-inflammatory signaling cascades that favor epithelial–mesenchymal transition and tumor initiation (38, 39). However, these metabolic circuitries, particularly the bile-acid and tryptophan–kynurenine axes, have been more comprehensively characterized in metabolic disease, colorectal cancer, and inflammatory bowel disorders, indicating that their mechanistic roles in PDAC require further validation (40–43).

Recent mechanistic studies further reveal that chronic inflammation reshapes the tumor immune microenvironment through myeloid-cell reprogramming. Specifically, CSF1R^+^ tumor-associated macrophages suppress cytotoxic CD8^+^ T-cell infiltration and effector function, generating an immunosuppressive niche that supports tumor persistence. Targeted depletion of this subset restores antitumor T-cell activity and reduces tumor burden, highlighting the CSF1/CSF1R axis as a promising immunotherapeutic target in PC (44).

Beyond immune modulation, the gut microbiome exerts systemic effects through the microbiota-gutbrain axis. Microbial species such as *Lactobacillus* and *Bifidobacterium* produce key neurotransmitters, including γ-aminobutyric acid (GABA), serotonin, and dopamine, that influence both vagal signaling and immune tone (45). This bidirectional communication system integrates neural, endocrine, and immune signals, modulating not only emotional and behavioral responses but also inflammatory pathways (46). Nevertheless, most mechanistic insights into microbiota-derived neurotransmission originate from neuropsychiatric, metabolic, and gastrointestinal disease models, with only indirect evidence linking these neuromodulatory pathways to PDAC biology. Consequently, their contribution to pancreatic tumorigenesis should be regarded as emerging rather than definitively established (47).

Shifts in the balance of dominant phyla, such as *Firmicutes* and *Bacteroidetes*, can significantly impact host metabolism and immune regulation, contributing to the development of metabolic syndrome, chronic inflammation, and tumorigenesis (48). Lastly, these findings position the gastrointestinal microbiome as a central but heterogeneously validated driver of PC pathogenesis, combining well-established inflammatory mechanisms with several promising neuroendocrine and metabolic pathways.

## Urogenital microbiome

Traditionally viewed as a sterile fluid and passive filtrate of systemic physiology, urine is now recognized as a dynamic biofluid capable of capturing early molecular changes associated with disease processes, including PDAC (49) (Figure 1). Emerging evidence suggests that urine harbors a range of biomarkers, including metabolites linked to glucose dysregulation, chronic inflammation, and cancer-associated cachexia, that can distinguish PDAC patients from healthy individuals even before the onset of clinical symptoms (50). One promising avenue involves urinary exosomal microRNAs, which are protected within lipid vesicles and reflect oncogenic signals emanating from the TME. These vesicle-encapsulated molecules exhibit remarkable stability, making them attractive candidates for non-invasive early detection (51). Although the composition of the urinary microbiota itself remains underexplored in the context of PDAC, the molecular cargo of urine may indirectly reflect host-microbiota interactions or microbial-driven metabolic shifts that contribute to tumor initiation and progression (52).

Crucially, urine does not merely mirror metabolic disturbances; it may also encode immunological and microbial alterations relevant to cancer biology. This broad molecular representation has led to the development of multiplexed biomarker panels, which integrate proteins, metabolites, and non-coding RNAs (53–55). Such combinatorial strategies consistently outperform single-analyte diagnostics in sensitivity and specificity, offering a robust approach to address the clinical challenges posed by PC, a malignancy characterized by marked heterogeneity and rapid progression (56). Collectively, urine has emerged as a powerful non-invasive matrix capable of capturing early molecular signals of malignancy in PC, supporting its integration into risk-stratification workflows.

### Urogenital microbiome and host-microbial interactions

Once considered sterile, the urinary and genital tracts are now recognized as dynamic microbial ecosystems essential for maintaining mucosal homeostasis and immune defense. Advanced sequencing technologies, particularly 16S rRNA and metagenomic approaches, have revealed diverse microbial consortia inhabiting these niches, collectively referred to as the *urogenital microbiome* or *urobiome*. These microorganisms, though present in low biomass, engage in intricate metabolic and immunological cross-talk with the host, influencing health, disease susceptibility, and therapeutic outcomes (29, 57).

The discovery that the urinary tract is not sterile has transformed urology and microbiome science. High-throughput sequencing and enhanced quantitative urine culture (EQUC) methods demonstrated viable microbial communities distinct from contamination by skin or vaginal flora. Predominant genera such as *Lactobacillus*, *Streptococcus*, *Gardnerella*, *Prevotella*, *Corynebacterium*, *Veillonella*, and *Escherichia-Shigella* constitute the core urinary microbiota, varying according to sex, age, and hormonal status (19, 29, 58–60). In women, *Lactobacillus*-dominated communities exert protective functions by producing lactic acid, hydrogen peroxide, and bacteriocins that maintain acidic pH and inhibit uropathogen adhesion. In contrast, dysbiosis, characterized by the overgrowth of *Gardnerella vaginalis*, *Ureaplasma urealyticum*, or *Escherichia coli*, is linked to urinary tract infections (UTIs), urgency urinary incontinence, interstitial cystitis, and elevated inflammatory signaling (29).

Host-microbial interactions in the urogenital tract are primarily mediated by mucosal immunity and biochemical communication. The urothelium expresses pattern-recognition receptors (PRRs) such as Toll-like receptors (TLRs), which recognize microbial-associated molecular patterns (MAMPs) and trigger innate immune cascades. These interactions activate antimicrobial peptide release and cytokine secretion, promoting a state of controlled immune vigilance. Symbiotic bacteria can also modulate host gene expression through short-chain fatty acids (SCFAs), indoles, and bile acid derivatives that act as epigenetic and metabolic regulators. For instance, SCFAs and deoxycholic acid regulate Th1/Th2 balance and macrophage polarization, thereby influencing local immune tolerance and inflammatory resolution. Dysregulation of these pathways can predispose individuals to recurrent UTIs or chronic inflammatory disorders (61, 62).

At the ecological level, quorum sensing and metabolite exchange underpin microbial community stability. Recent metabolomic-metagenomic integration has identified co-regulated networks linking specific metabolites to microbial taxa in the female urinary tract. Protective *Lactobacillus* species were associated with lipids and amino acids maintaining epithelial barrier integrity, while pathogenic *E. coli* correlated with elevated polyamines such as putrescine and pro-inflammatory lipids. Deoxycholic acid emerged as a prognostic metabolite for recurrent UTI, reflecting metabolic shifts toward dysbiosis and immune suppression (62).

Mechanistically, cross-talk between the urinary microbiome and systemic physiology extends beyond local infection. The gut-urogenital axis links intestinal dysbiosis to urinary tract inflammation via translocation of microbial metabolites and immune mediators. In particular, bacterial lipopolysaccharides and tryptophan-kynurenine pathway metabolites modulate systemic immunity, affecting conditions such as bladder cancer and prostate inflammation. Moreover, gut-derived microbial metabolites can regulate hormonal pathways, including estrogen metabolism, which influences vaginal and urinary microbial composition and postmenopausal susceptibility to rUTI (62, 63).

Emerging research further implicates the urogenital microbiota in carcinogenesis. Dysbiosis can promote chronic inflammation, oxidative stress, and immune evasion, fostering tumorigenic microenvironments in the bladder, prostate, and kidneys. In urothelial carcinoma, reduced microbial diversity and enrichment of *Fusobacterium nucleatum*, *Acinetobacter*, and *Corynebacterium* species correlate with increased cytokine production and DNA damage. Conversely, commensal *Lactobacillus* and *Streptococcus* species exhibit potential anti-tumor effects by producing anti-inflammatory metabolites and modulating immune checkpoints. Integrative analyses suggest that urogenital dysbiosis and gut microbiome alterations jointly influence immune checkpoint inhibitor (ICI) response in bladder and renal cancers, with SCFAs enhancing Th1 polarization and tumor immunosurveillance (61, 63, 64).

Although extensive evidence links the gut, oral, and intrapancreatic microbiota to PDAC, the urogenital niche remains an underexplored microbial compartment in this malignancy. Recent evidence indicates that microbial colonization within pancreatic tissue is not merely contaminant but metabolically active, shaping immune surveillance and clinical outcomes in PDAC patients (65, 66).

Intratumoral and intestinal dysbiosis have been shown to modulate chemotherapeutic efficacy and immune responses through microbial metabolites and inflammatory signaling pathways (67, 68). The microbiome is therefore emerging as both a biomarker and a therapeutic target across gastrointestinal malignancies, including PDAC, where altered microbial signatures correlate with disease stage and prognosis (69). Microbial metabolites act as key mediators linking host-microbial metabolic interactions to oncogenic signaling cascades. Moreover, fecal microbiota transplantation studies reinforce the therapeutic potential of microbiome modulation in PDAC (70, 71).

Complementarily, urinary biomarker research has revealed that urine can function as a non-invasive reservoir of molecular indicators for early PDAC detection, suggesting that microbial or metabolic products from distal compartments may reach the urinary tract (50). This supports the broader concept of a microbiome-tumor axis that integrates cross-compartmental microbial trafficking and systemic immune-metabolic regulation. However, no empirical study has yet characterized the urogenital microbiome in PDAC, representing a significant gap in the *oncobiome* field. The established immunometabolic influence of urogenital microbiota in other genitourinary cancers and the identification of urinary biomarkers in PDAC provide a strong rationale to investigate whether microbial communities from the urinary or reproductive tracts contribute to systemic inflammation, metabolite circulation, or immune dysregulation in pancreatic tumorigenesis (64, 72). Together with previous findings on urinary biomarkers, these insights suggest that urine may serve not only as a molecular biospecimen but also as a reflective medium of systemic and local microbial dynamics associated with PDAC.

Methodological advances have been essential for defining these interactions. Low microbial biomass in urine samples presents unique challenges, requiring stringent contamination control and optimized sequencing pipelines. Enhanced protocols, such as EQUC, catheterized sampling, and multi-omic integration, enable differentiation between resident microbiota and contaminants while revealing functional pathways associated with immunity and metabolism. Standardization across studies remains crucial for reproducibility and for translating microbial signatures into clinical diagnostics (73, 74).

Recent comprehensive analyses of over 1,000 individuals demonstrated gender- and age-dependent microbial clustering into distinct “urotypes,” each dominated by characteristic taxa such as *Lactobacillus*, *Prevotella*, or *Corynebacterium* (19). These urotypes exhibit unique immunometabolic profiles and may define baseline microbial “set points” of urinary health. Shifts in these profiles could therefore serve as early indicators of pathological states or treatment outcomes. The concept of the *oncobiome*, microbial genetic and metabolic contributions to carcinogenesis, has emerged as a key framework linking urinary microbiota to urological cancers, suggesting novel avenues for biomarker discovery and therapeutic modulation (63, 64).

### Urinary biomarkers and non-invasive diagnostics

Over the past decade, the search for reliable non-invasive diagnostic tools in PDAC has increasingly focused on urine as a valuable biofluid. Its stability, ease of collection, and capacity to capture both systemic and tumor-specific molecular changes make it an attractive matrix for biomarker discovery. Notably, multiple studies have shown that urinary transcripts and proteins, particularly lymphatic vessel endothelial hyaluronan receptor 1 (LYVE-1), regenerating islet-derived protein 1 alpha/beta (REG1A/REG1B), and trefoil factor 1 (TFF1), are significantly elevated in PDAC and can effectively distinguish early-stage tumors from benign pancreatic or hepatobiliary conditions with high diagnostic accuracy. A combined three-marker panel incorporating LYVE-1, REG1A, and TFF1 achieved sensitivities of up to 96% and specificities approaching 100%, representing a major step forward toward early, non-invasive detection of PDAC (75–77).

Building on these findings, the same group validated the diagnostic value of this urinary biomarker panel (PancRISK) in large, multicentric and pre-diagnostic cohorts, revealing its capacity to detect PDAC up to 2 years before clinical diagnosis. When combined with plasma CA19-9, the panel achieved an AUC of 0.89 for early-stage detection and maintained strong discriminative power for cases diagnosed up to 2 years later (77). This longitudinal validation supports the integration of urinary markers into risk-stratification algorithms, particularly for high-risk individuals such as those with chronic pancreatitis or new-onset diabetes.

Recent investigations have expanded the molecular spectrum of urinary biomarkers by incorporating inflammatory mediators and exosomal cargo. For instance, urinary C-reactive protein (uCRP) exhibited significant diagnostic performance when used alone or in combination with LYVE-1, REG1B, and TFF1, improving sensitivity and AUC values compared with plasma CA19-9 alone (78). Exosome-derived molecules have also gained attention as robust carriers of tumor-specific information. Exosomal RNA and protein signatures in blood and urine mirror oncogenic pathways, including immune escape, angiogenesis, and metabolic reprogramming, offering a stable medium for liquid biopsy applications (79). The urinary exosome compartment provides a particularly valuable window into pancreatic tumor biology, as small RNAs and microRNAs encapsulated within vesicles remain protected from degradation and can be detected non-invasively (80).

MicroRNA-based assays are currently among the most advanced urinary diagnostic strategies for PDAC. A multicenter study identified a urinary extracellular-vesicle miRNA panel capable of detecting PDAC across all stages, with AUC values exceeding 0.96 and sensitivity above 90% in early-stage disease (80). These miRNAs reflected tumor and microenvironmental expression patterns, supporting their biological relevance as surrogates of tumor activity. Similarly, serum-based miRNA sequencing combined with artificial intelligence models achieved near-perfect discrimination between PDAC and controls (AUC = 0.99), demonstrating the potential of machine-learning-enhanced molecular profiling for clinical translation (81).

Beyond nucleic acids, metabolomic profiling of urine provides complementary insight into PDAC pathophysiology. Systematic reviews have identified recurrent alterations in amino acid and lipid pathways, including changes in glucose, lactate, and bile acid metabolism, which reflect the metabolic reprogramming characteristic of pancreatic carcinogenesis (77). Specific urinary metabolites such as deoxycholic acid, identified in urinary biochemical ecology studies, have been linked to microbiome-associated metabolic disturbances and could serve as prognostic indicators for disease progression or recurrence (62).

Parallel to these advances, novel serum markers continue to emerge that complement urinary diagnostics. Laminin γ2 monomer (LG2m) has recently been identified as a circulating biomarker with higher diagnostic accuracy than CEA and CA19-9, achieving an AUC = 0.88 and providing prognostic value for treatment outcomes. Although serum-based, LG2m illustrates the importance of integrating multi-fluid biomarker systems where urinary, serum, and exosomal components provide a unified diagnostic landscape. Such multi-analyte panels, when combined with clinical parameters and imaging data, could redefine early PDAC screening strategies (82).

Despite encouraging results, the clinical application of urine-based biomarkers for PDAC requires cautious interpretation. The three-protein panel (LYVE1, REG1B, TFF1), showed very high discriminative performance in retrospective samples: in the discovery/validation sets they reported an area under the ROC curve (AUC) of 0.97 (95% CI 0.94–0.99), with sensitivity and specificity reportedly >85% for early-stage PDAC vs. controls (83).

However, in subsequent longitudinal validation using pre-diagnostic urine samples (PancRISK), although still robust, performance was attenuated: the urine-based score combined with plasma CA19-9 achieved an AUC of 0.89 for early-stage PDAC detection, with sensitivity 72% and specificity 90% up to 1 year before diagnosis, a decline compared to the case-control setting (77).

Additional work indicates that integrating inflammatory markers (e.g., urinary C-reactive protein, uCRP) into the original panel enhances diagnostic accuracy (reported AUC ∼ 0.92), but these enhancements remain preliminary, and independent validation across diverse cohorts and analytical platforms is still lacking (77). Urine-derived molecular assays thus represent a compelling yet technically fragile frontier in PDAC detection, where analytical rigor and multicentric harmonization will determine their eventual clinical utility.

## Oral microbiome

Homeostasis between the human host and its resident oral microbiota is essential for maintaining normal physiological function (Figure 1). The oral microbiome, one of the most diverse microbial ecosystems in the body, is shaped by a range of factors, including age, sex, oral hygiene, diet, and immune status. Dominant genera commonly identified in the oral cavity include *Streptococcus*, *Haemophilus*, *Leptotrichia*, *Porphyromonas*, *Prevotella*, *Propionibacterium*, *Staphylococcus*, *Veillonella*, and *Treponema* (30). Constantly exposed to exogenous microorganisms through breathing, eating, and drinking, the oral cavity functions as a dynamic interface between host immunity and the external environment (84). This continuous microbial influx complicates the distinction between resident and transient species. Nevertheless, accumulating evidence indicates that specific oral pathogens can disseminate beyond the oral cavity, via saliva, biliary pathways, hematogenous spread, or lymphatic transport, to distant organs such as the gastrointestinal tract and pancreas, where they may alter local microenvironments and promote tumorigenesis (85).

In a Chinese cohort comparing the oral microbiota of individuals with PC, benign pancreatic disease (BPD), and healthy controls (HC), significant compositional differences were observed. *Proteobacteria* dominated the oral microbiota of healthy and BPD groups, while *Bacteroidetes* predominated in the PC group (85, 86). At the family level, *Leptotrichiaceae*, *Actinomycetaceae*, *Lachnospiraceae*, *Micrococcaceae*, *Erysipelotrichaceae*, *Coriobacteriaceae*, and *Moraxellaceae* were enriched in PC, whereas *Porphyromonadaceae*, *Campylobacteraceae*, and *Spirochaetaceae* were depleted (87). Nonetheless, the relative abundance of these taxa exhibits substantial variation across geographic cohorts, sample sources, and sequencing methodologies. These inconsistencies highlight the necessity of standardized analytical frameworks and cross-cohort validation to ensure reproducibility and accurate interpretation of oral microbiome-cancer associations (88, 89). Notably, PC patients exhibited elevated levels of pro-inflammatory and immunosuppressive oral pathogens such as *P. gingivalis* and *A. actinomycetemcomitans*, which activate inflammatory cascades and impair immune surveillance (90). Conversely, reductions in commensal taxa like *Leptotrichia* and *Fusobacterium nucleatum* suggest that both pathogenic overgrowth and loss of beneficial species may drive carcinogenic processes (87).

## Intrapancreatic microbiome

Once considered a sterile site, the pancreas is now recognized as a host to a distinct, low-biomass microbiome that becomes enriched and metabolically active during PC progression. However, this assertion must be interpreted with caution given the well-documented challenge of distinguishing genuine intrapancreatic microbial signals from reagent or procedural contaminants inherent to low-biomass tissues. Such contamination risks can confound sequencing-based analyses, underscoring the need for rigorous negative controls, contamination-aware bioinformatic filtering, and functional validation to confirm true microbial presence and activity (91, 92). Evidence indicates that both commensal and pathogenic microorganisms can translocate from the gut or oral cavity to the pancreatic tissue, where they reprogram the TME through immune modulation, inflammatory signaling, and metabolic interactions (93, 94).

In PDAC, the intrapancreatic microbiota is predominantly composed of taxa from the phyla Proteobacteria, Firmicutes, Bacteroidetes, and Actinobacteria, with recurrent enrichment of genera such as *Fusobacterium*, *Pseudomonas*, *Enterobacter*, *Escherichia*, *Klebsiella*, *Streptococcus*, *Bifidobacterium*, and fungal species such as *Malassezia* (22, 95). These microbial populations contribute to tumorigenesis by promoting both innate and adaptive immune suppression, expanding myeloid-derived suppressor cells (MDSCs) and regulatory T cells, while attenuating cytotoxic CD8^+^ T-cell activity (36). Notably, *F. nucleatum* and *Malassezia* spp. accelerate PDAC progression by activating complement pathways, driving NF-κB dependent inflammation, and enhancing stromal fibrosis (21, 96).

In contrast, long-term PDAC survivors display a unique microbial signature characterized by higher alpha diversity and enrichment of *Pseudoxanthomonas*, *Saccharopolyspora*, and *Streptomyces*, taxa linked to robust antitumor immune responses and improved overall survival (22). This compositional heterogeneity underscores the potential prognostic value of intrapancreatic microbial profiles.

Beyond tumorigenesis, intratumoral bacteria influence therapeutic efficacy through metabolic interactions and immune modulation. Certain taxa are capable of enzymatically modifying anticancer drugs, thereby contributing to variable treatment responses and chemoresistance in PDAC (21). Moreover, microbiome-driven transcriptional reprogramming within the tumor compartment promotes epithelial-to-mesenchymal transition and favors basal-like PDAC subtypes, which are associated with poor prognosis (95). Deep-sequencing of intratumoral microbiota has recently delineated *core microbial consortia* associated with stromal remodeling and therapy response. Enrichment of *Pseudomonas aeruginosa* and *Klebsiella oxytoca* correlates with elevated IL-1β and TGF-β signaling, potentiating desmoplasia and immune exclusion (97). Conversely, tumors harboring *Bifidobacterium longum* and *Streptococcus mitis* show enhanced MHC-I expression and improved responsiveness to gemcitabine (98).

Altogether, these insights position the intrapancreatic microbiome as a dynamic and therapeutically relevant axis in PDAC biology, where precision modulation, via antibiotics, probiotics, or fecal microbiota transplantation (FMT), holds potential to enhance chemotherapeutic efficacy, sensitize tumors to immunotherapy, and reshape future paradigms in PC management (94, 99, 100).

## Microbiome bacteria associated with pancreatic cancer

Dysbiosis refers to an imbalance in the composition and function of the commensal microbiota, disrupting host-microbe equilibrium and predisposing to disease (101). In healthy individuals, the microbiota plays a central role in maintaining immune homeostasis, regulating metabolism, and suppressing opportunistic pathogens (102). However, when microbial balance is disturbed, a cascade of pro-inflammatory responses can be initiated. This inflammatory milieu disrupts host biochemical pathways and immune defenses, creating a permissive environment for disease progression and oncogenesis (103). In PDAC, dysbiosis-driven chronic inflammation has been linked to enhanced infiltration and activation of CD8^+^ T cells, an immune response that paradoxically coexists with an immunosuppressive TME (104).

Specific microbial taxa have been shown to influence both the immune landscape and therapeutic response in PDAC. Certain *Gammaproteobacteria* harbor cytidine-deaminase enzymes that metabolize gemcitabine into inactive derivatives, illustrating how microbial enzymatic activity can directly influence pharmacologic efficacy and therapeutic outcomes in PDAC (105). Another key oncomicrobe, *Fusobacterium nucleatum*, a Gram-negative anaerobe typically resident in the oral cavity, has been detected in both colorectal and pancreatic tumors. Under pathological conditions, it disseminates systemically, promoting tumor growth, immune evasion, and epigenetic silencing of tumor suppressor genes through hypermethylation and microsatellite instability. Mechanistically, *F. nucleatum* enhances secretion of IL-8 and CXCL1 and disrupts the centrosomal protein CEP55, facilitating malignant transformation (106, 107). *Enterococcus faecalis*, a Gram-positive facultative anaerobe, also emerges as a relevant organism in PDAC. Normally residing in the gut, *E. faecalis* has been detected in pancreatic juice and tissue of patients with suspected malignancy. Its resistance to alkaline environments enables survival in the pancreatic duct, where it may invade host cells via endocytosis, supporting its role as a potential microbial biomarker for PC (108).

While classically linked to gastric carcinogenesis, *Helicobacter pylori* is increasingly associated with pancreatic tumorigenesis. Two primary mechanisms have been proposed: (i) chronic infection leading to depletion of antral D-cells and reduced somatostatin, which in turn elevates secretin and bicarbonate secretion, inducing pancreatic ductal hyperplasia; and (ii) bacterial overgrowth and gastric hypoacidity fostering N-nitrosamine formation, potent carcinogens that cause DNA damage and neoplastic transformation (10, 109).

Another notable oral pathogen, *P. gingivalis*, has also been linked to PDAC. Primarily associated with periodontitis, *P. gingivalis* can invade the pancreas and trigger systemic inflammatory responses (110). Once established in the pancreatic tissue, it releases peptidyl-arginine deiminase (PAD), which promotes the formation of neutrophil extracellular traps (NETs). These, in turn, stimulate the release of pro-tumorigenic chemokines such as CXCL1, CXCL2, and neutrophil elastase, contributing to fibrosis, metastasis, immune evasion, and enhanced tumor proliferation (12, 33, 111).

Together, these microbial communities orchestrate chronic inflammation, immune dysregulation, epigenetic remodeling, and metabolic interference with chemotherapy, progressively transforming the TME into an immune-suppressive, pro-tumorigenic niche that underscores the microbiome’s role as a mechanistic driver and clinically actionable target in PDAC (112, 113).

## Inflammatory mechanisms mediated by the microbiome in pancreatic cancer

The human gastrointestinal microbiota, primarily composed of four dominant phyla: *Firmicutes, Bacteroidetes, Actinobacteria*, and *Proteobacteria*, plays a fundamental role in the development and modulation of both the innate and adaptive immune systems (114). Disruptions to this microbial balance, or dysbiosis, can lead to chronic inflammation, genomic instability in pancreatic acinar cells, and impaired antitumor immune surveillance, key factors in the pathogenesis of PDAC (37).

Epidemiological data consistently implicate the oral microbiota as a contributor to PC risk. Elevated oral abundance of *P. gingivalis* (OR 1.6; 95% CI 1.15–2.22) and *A. actinomycetemcomitans* (OR 2.22; 95% CI 1.16–4.18) has been significantly associated with increased PDAC susceptibility (90). Beyond individual species, a meta-analysis of eight observational studies reported a higher incidence of PC in individuals with periodontitis (RR 1.74; 95% CI 1.41–2.15) and in edentulous subjects (RR 1.54; 95% CI 1.16–2.05), independent of confounders such as age, smoking, diabetes, or alcohol use (115). Expanding on these associations, pooled data from 14 observational studies (*n* = 1,276; 285 PDAC, 342 chronic pancreatitis, 649 controls) revealed enrichment of *Actinomyces*, *Bifidobacterium*, *Veillonella*, and *Escherichia-Shigella*, alongside depletion of *Firmicutes* in PDAC cohorts (116). Similarly, retrospective biliary tract analyses identified dominant Gram-negative taxa, *E. coli*, *Klebsiella* spp., and *Pseudomonas* spp., in PDAC but not in extra-pancreatic malignancies, suggesting their involvement in sustaining localized inflammatory and pro-tumorigenic signaling (117).

The well-recognized association between chronic pancreatitis and PC further underscores the oncogenic role of persistent inflammation (118). Inflammation-induced remodeling of the TME drives PDAC progression. The PDAC TME is dominated by a dense desmoplastic stroma, constituting up to 90% of the tumor mass, rich in pancreatic stellate cells, fibroblasts, macrophages, neutrophils, regulatory T cells, and myeloid-derived suppressor cells (MDSCs). This fibrotic niche, defined by hypoxia, acidity, and poor perfusion, facilitates immune evasion and therapeutic resistance (119). Elevated systemic concentrations of IL-6, IL-8, and IL-10 have been linked to reduced overall survival in PDAC patients (HR 0.204, 0.303, 0.336; all *p* < 0.05), highlighting their prognostic significance (120). Mechanistically, microbial dysbiosis triggers Toll-like receptor (TLR) activation in monocytes, promoting their M2 macrophage polarization and IL-10 secretion, thereby suppressing cytotoxic T-cell responses. TLR-mediated signaling also supports MDSC expansion and Th2 differentiation while impairing Th1 and CD8^+^ T-cell activity (121). Key cytokines within the TME, including GM-CSF and CXCL1, further sustain metastatic potential and chemotherapy resistance (111). These findings delineate a microbiome-immune axis that orchestrates chronic inflammation, immune tolerance, and stromal remodeling in PDAC. Microbial dysbiosis emerges not merely as a byproduct of disease but as a central driver of tumor-promoting immunity, positioning the microbiome as a promising frontier for biomarker discovery and targeted intervention in PC (122).

Integrative analyses underscore that the microbiome-immune-stromal triad governs PDAC evolution. Single-cell transcriptomic and metagenomic coupling demonstrated that microbial lipopolysaccharide and peptidoglycan signatures activate tumor-associated fibroblasts, inducing CXCL12-mediated immune sequestration and chemoresistance (38). This microbial-stromal axis adds a new mechanistic layer to chronic inflammation and may explain why anti-inflammatory interventions alone yield limited efficacy (97).

## Therapeutic response and the microbiome

Current therapeutic strategies for PC primarily involve surgical resection, when feasible, in combination with adjuvant systemic chemotherapy, with or without radiotherapy. Commonly employed chemotherapy regimens include gemcitabine monotherapy, gemcitabine plus capecitabine or cisplatin, and folfirinox (a combination of fluorouracil, leucovorin, irinotecan, and oxaliplatin) (123). Despite these interventions, treatment outcomes remain poor, with minimal improvement in long-term survival. Both chemotherapy and radiotherapy disrupt intestinal mucosal integrity, promoting dysbiosis characterized by loss of microbial diversity and functional imbalance. This microbial disruption aggravates gastrointestinal toxicity, manifested as nausea, vomiting, and diarrhea, and impairs anti-tumor immunity, further limiting therapeutic efficacy (124, 125).

Bacterial cytidine deaminase converts gemcitabine into its inactive metabolite, 2′,2′-difluorodeoxyuridine, thereby reducing intracellular drug availability and fostering chemoresistance. This mechanism, identified in *Gammaproteobacteria* such as *E. coli* and *Klebsiella pneumoniae*, exemplifies the metabolic interplay between tumor-associated microbes and pharmacologic response. Translational evidence links elevated microbial cytidine-deaminase activity with diminished chemotherapeutic efficacy in PDAC, underscoring the potential of microbiome-targeted strategies to restore drug sensitivity and improve patient outcomes (21, 105, 126).

Certain gut taxa counteract these adverse effects by enhancing immune surveillance and metabolic homeostasis. *Bifidobacterium* and *Faecalibacterium*, for instance, ferment dietary substrates into SCFAs such as butyrate, which exert potent anti-inflammatory and immunomodulatory functions (127). Butyrate enhances cytotoxic responses by stimulating CD4^+^ and CD8^+^ T cells to produce interferon-γ (IFN-γ), facilitating tumor-infiltrating lymphocyte recruitment and malignant cell clearance (128). Conversely, intestinal dysbiosis can attenuate chemotherapy responsiveness. In a murine model, Kesh et al. showed that diabetic mice with altered gut microbiota developed resistance to gemcitabine/paclitaxel compared to non-diabetic controls, underscoring the microbiome’s influence on pharmacodynamics (129).

The PDAC immune landscape is further shaped by checkpoint pathways such as PD-1/PD-L1 and CTLA-4, which suppress T-cell activity. Immune checkpoint inhibitors (ICIs) restore anti-tumor immunity by blocking these inhibitory signals (130). Notably, antibiotic-induced microbial depletion increased PD-L1 expression and enhanced ICI efficacy in PDAC mouse models (21). Yet some microorganisms foster chemoresistance. *E. coli* and *Saccharomyces cerevisiae* express cytidine deaminase, which inactivates gemcitabine by converting it into 2′,2′-difluorodeoxyuridine (126). In a cohort of 712 early-stage PDAC patients, antibiotic administration within 1 month of chemotherapy initiation improved overall survival by 37% and cancer-specific survival by 30%, though at the cost of increased treatment-related toxicity (131). Similarly, concurrent antibiotic use in metastatic PDAC was associated with higher rates of hematological toxicity, including anemia, thrombocytopenia, leukopenia, and neutropenia (132).

Microbiome modulation has therefore emerged as a promising adjunctive strategy. Probiotic-derived metabolites such as ferrichrome from *Lactobacillus casei* trigger p53-dependent apoptosis and DNA fragmentation in chemoresistant cancer cells, reducing tumor burden in murine models (133). Likewise, butyrate produced by *Firmicutes* preserves epithelial barrier integrity and suppresses neoplastic transformation in gemcitabine-treated mice (134). Another promising approach is FMT. In a seminal study, Riquelme et al. transplanted fecal samples from long-term (>5 years) and short-term (<5 years) PDAC survivors into murine models. Mice receiving microbiota from long-term survivors exhibited significantly reduced tumor growth, suggesting that specific microbial configurations may contribute to better clinical outcomes (22). Building on these findings, a phase I clinical trial (NCT04975217) is currently evaluating the safety and efficacy of FMT in PDAC patients undergoing surgical resection (71). While preclinical studies highlight the therapeutic promise of microbiota modulation, translation into routine clinical practice remains limited, and robust, randomized trials are essential to determine efficacy, safety, and durability of response in humans.

Building on this rationale, integrative immunotherapy platforms now combine checkpoint modulators, oncolytic or vaccine approaches, and engineered immune cells within microbiome-aware frameworks. These strategies exploit microbial influences on antigen presentation, cytokine balance, and myeloid polarization, aligning with chronotherapy-based and aI-assisted response modeling (135). However, some mechanistic frameworks, such as the proposed neuro-immune–microbiota axis, are supported largely by studies in colorectal, gastric, and neuroinflammatory disease models rather than pDAC-specific evidence. in these systems, neuropeptides (e.g., CGRP, substance P, VIP) reshape fibroblast activation, myeloid behavior, and lymphocyte trafficking, processes that may be relevant to PDAC but remain insufficiently validated in this tumor type (136, 137). Parallel advances nonetheless suggest that neuronal stress and neuromodulatory signaling could influence stromal architecture and immune tone. Accordingly, we present the neuro-immune–microbiota axis as an emerging, hypothesis-generating concept that warrants targeted mechanistic investigation before clinical integration in PDAC (17).

## Methodological considerations and limitations

### Contamination challenges in low-biomass microbiome studies

Low-biomass microbiome research, including studies of pancreatic tissue, cystic lesions, and bile, faces persistent obstacles related to contamination that can easily overshadow genuine microbial signals. In these environments, the host-to-microbe DNA ratio often exceeds 10^6^:1, meaning that even trace exogenous DNA from reagents, air, or cross-sample contact may generate false positives and distort community profiles. These challenges are particularly significant in pancreatic cancer research, where the existence of a stable “intrapancreatic microbiome” remains under debate (91, 92).

Quantitative analyses have demonstrated that, even when strict decontamination protocols are applied, residual contamination can still bias statistical outcomes. When more than ten contaminant taxa are present, false positives in differential abundance analysis increase substantially, affecting downstream interpretation. However, when validated protocols and internal negative controls are employed, contamination exerts minimal influence on overall diversity metrics. Internal negative controls are thus preferred over universal “contaminant lists,” which have shown high inconsistency between studies and lack reproducibility (91).

The debate over contamination is particularly evident in pancreatic research. Studies using advanced sequencing and decontamination workflows have shown that microbial reads in pancreatic tissue are extremely sparse and often comparable to those in negative controls. In analyses of intraductal papillary mucinous neoplasms, microbial signatures were indistinguishable from sterile syringes collected in the same surgical setting, indicating that prior invasive procedures or environmental exposure likely introduced bacterial DNA rather than reflecting true colonization (92, 138).

Low-biomass studies also suffer from host-DNA misclassification, database contamination, and batch effects that amplify artifactual patterns. Computational pipelines have shown that the majority of microbial reads in blood or tissue datasets may derive from misannotated host sequences or residual reagent DNA. The use of machine learning and reference-based filtering has improved precision, yet even the most robust bioinformatic pipelines can be compromised by cross-contamination among wells or by sequencing run variability (139, 140).

Evidence from pancreatic cancer further illustrates these limitations. Analyses of pancreatic tumors and adjacent tissues revealed uniformly low bacterial biomass, comparable to negative controls, suggesting that much of the detected DNA arises from contamination rather than viable microorganisms. These findings contrast with earlier preclinical work that attributed chemotherapy resistance to *Gammaproteobacteria* capable of metabolizing gemcitabine. Similarly, meta-analyses linking tumor-associated bacteria to therapeutic outcomes must be interpreted cautiously, as low microbial loads and sequencing artifacts can mimic true microbial presence. While some studies reported bacterial metabolites influencing chemotherapy efficacy, contamination remains a confounding factor that challenges reproducibility (141, 142).

Research addressing metabolic and environmental modifiers of pancreatic carcinogenesis has also acknowledged the complexity of low-biomass detection. Experimental models investigating obesity-driven changes in pancreatic microbiota have reported microbial shifts accompanying accelerated tumor progression, but these differences may reflect contamination from intestinal sources or amplification bias inherent to low microbial loads (143).

At the methodological level, international guidelines now provide clear frameworks to prevent and report contamination. The consensus emphasizes that contamination cannot be completely eliminated but must be minimized through procedural rigor at every step. Recommended measures include the use of single-use DNA-free consumables, ethanol and nuclease decontamination, ultraviolet sterilization of laboratory surfaces, and inclusion of multiple negative and positive controls throughout sampling and library preparation. Transparent reporting of all decontamination and sequencing procedures is essential to ensure reproducibility and data comparability (140, 144).

### Cohort variability and sequencing platform bias

Inter-study variability remains a fundamental challenge in microbiome-oncology, particularly in low-biomass environments such as the pancreatic tumor microenvironment. Differences in cohort composition, geographical origin, disease stage, biospecimen type, and clinical handling, together with technical discrepancies in DNA extraction, library preparation, and sequencing platform, create substantial heterogeneity that can obscure genuine microbial signals. Comparative meta-analyses reveal that even studies targeting the same malignancy often produce discordant microbial signatures, largely driven by methodological inconsistencies and platform-dependent bias (88, 89).

A major source of variability arises from sequencing methodology. Shallow shotgun metagenomics provides lower technical variation and greater taxonomic resolution than 16S rRNA sequencing, while maintaining cost efficiency for large-scale studies. However, both approaches remain susceptible to upstream biases during DNA extraction and amplification. Primer choice, GC-content bias, and library preparation can all influence the observed microbial community composition, underscoring that every step from sample collection to sequencing may introduce systematic distortion (89, 145).

Sequencing platform bias also propagates through downstream bioinformatics pipelines. Even when standardized reference databases are applied, subtle algorithmic variations in classification can yield divergent abundance profiles. Batch effects further compound this variability by introducing non-biological differences across sequencing runs or laboratories; correction models such as ConQuR mitigate these effects using conditional quantile regression while preserving biological signals (146). Similarly, meta-analytic frameworks like Melody harmonize summary statistics across studies while accounting for compositionality and variable sequencing depth, thereby improving the generalizability of microbial signatures (147).

Cross-cohort variability remains equally problematic. Large-scale pooled analyses, such as the 18-cohort meta-analysis of 3,741 stool metagenomes in colorectal cancer, have shown that prediction models trained in one dataset frequently underperform in others, even when identical analytical pipelines are applied. Microbial risk scores developed in one population often lose discriminatory power when transferred to other cohorts, reflecting strong geographic and cohort-specific confounding (88, 148).

In pancreatic cancer research, this issue is particularly pronounced. Differences in tissue acquisition methods, such as endoscopic ultrasound (EUS)-guided biopsy versus surgical resection, and sequencing modality can profoundly affect transcriptional and cellular composition profiles, influencing inferred tumor microenvironmental states (149). Pre-analytical variation and normalization strategies also significantly alter cfRNA biomarker detection in PDAC, reinforcing that handling and sequencing consistency are crucial for reliable cross-cohort biomarker validation (150).

Efforts to mitigate these discrepancies through database harmonization are exemplified by the GMrepo v3 initiative, which curated nearly 119,000 microbiome datasets under uniform analytical pipelines and introduced the Marker Consistency Index (MCI) to quantify reproducibility across diseases. This initiative demonstrated that fewer than half of reported microbial biomarkers remain directionally consistent across independent projects, emphasizing the pervasive impact of technical and cohort heterogeneity. Complementarily, integrative genomic studies in PDAC have revealed that even non-coding mutational landscapes can differ between treated and untreated cohorts due to batch-related artifacts and analytical pipeline discrepancies (151, 152).

### Limitations of 16S rRNA compared to shotgun metagenomics

The 16S rRNA gene sequencing approach, although fundamental in microbiome studies, presents intrinsic limitations when compared to shotgun metagenomics. By amplifying specific hypervariable regions of a single conserved gene, 16S sequencing cannot differentiate closely related species or strains, many of which play distinct biological roles in carcinogenesis and immune modulation (153). The dependence on primer selection and amplification cycles introduces compositional bias, while the variability in 16S gene copy number across taxa distorts relative abundance estimates and complicates comparisons across cohorts. These limitations are exacerbated in pancreatic cancer research, where microbial biomass is extremely low, making the method highly susceptible to contamination and stochastic amplification errors (145, 154).

A critical shortcoming of 16S rRNA sequencing lies in its limited functional capacity. It relies on predictive algorithms such as PICRUSt or Tax4Fun to infer gene content and pathway activity, which often misrepresent the actual functional landscape of microbial communities. Shotgun metagenomics, in contrast, sequences all genetic material within a sample, enabling direct detection of microbial genes, virulence determinants, antibiotic resistance markers, and metabolic pathways (153, 155).

Comparative analyses have demonstrated that shotgun metagenomics identifies a broader and more accurate range of taxa than 16S sequencing. For instance, it captures rare or low-abundance species that exert significant metabolic or immunological influence on the tumor microenvironment (145). In PDAC cohorts, shotgun-based profiling has revealed microbial and viral biomarkers capable of distinguishing cancer from control samples with high reproducibility across geographic populations. Such cross-cohort consistency remains unachievable with 16S sequencing, which often produces variable results due to primer bias and insufficient resolution (155, 156).

16S sequencing also excludes large components of the microbiome, including fungi, viruses, and archaea, which collectively shape cancer-associated microbial networks. Shotgun metagenomics overcomes this limitation by simultaneously capturing multi-kingdom microbial DNA, thereby enabling integrative bacteriome-mycobiome-virome analyses (157). In biliary and pancreatic cancer studies, shotgun sequencing uncovered fungal enrichment and dysregulated pathways involving sphingolipid, bile acid, and fatty acid metabolism, highlighting functional cross-talk between bacterial and fungal communities that would remain invisible with 16S-based approaches (158).

Differences in quantitative accuracy are equally pronounced. While both methods can resolve high-level taxonomic patterns, shotgun metagenomics provides finer species- and strain-level resolution, improving ecological interpretation and clinical relevance. It has demonstrated superior accuracy in quantifying taxa abundance and detecting disease-related shifts in low-abundance microbial populations. These advantages are particularly meaningful in PDAC, where microbial heterogeneity and low biomass require both sensitivity and precision to detect biologically relevant taxa (156, 157).

From a technical perspective, 16S sequencing suffers from low reproducibility due to its dependence on primer design, read depth, and clustering algorithms. In contrast, shallow shotgun metagenomics achieves robust reproducibility with reduced technical variation while maintaining comprehensive taxonomic and functional coverage (159). Its lower cost compared to deep sequencing makes it a practical alternative for large-scale microbiome studies, combining resolution and feasibility. Furthermore, in clinical samples such as bile or pancreatic fluid, metagenomic next-generation sequencing (mNGS) exhibits greater pathogen detection sensitivity and higher diagnostic concordance than both culture and 16S methods (145, 160).

## Conclusion and future perspectives

The intricate interplay between the gastrointestinal, urogenital, oral, and intrapancreatic microbiomes and PC highlights a paradigm shift in our understanding of tumorigenesis, inflammation, and therapeutic resistance. Mounting evidence demonstrates that the microbiota is not merely a bystander but an active participant in modulating pancreatic oncogenesis, immune evasion, and chemoresistance (29, 86, 103). The gut microbiome influences PC onset and progression through dysbiosis, metabolite dysregulation, and immunosuppressive signaling, an axis that holds significant diagnostic and therapeutic potential (9, 10). Concurrently, urogenital microbiota signatures, particularly urinary metabolites and exosomal microRNAs, have emerged as promising non-invasive biomarkers (51). The oral microbiome, especially species such as *F. nucleatum* and *P. gingivalis*, has been associated with increased PC risk, suggesting potential utility in early detection through salivary or dental microbiota screening (90). Notably, the pancreatic TME harbors a low-biomass but metabolically active intratumoral microbiota, composed mainly of *Fusobacterium*, *Pseudomonas*, *Klebsiella*, *Escherichia*, *Streptococcus*, *Bifidobacterium*, and *Malassezia* species, which promotes immune suppression, modulates antitumor immunity, and inactivates drugs like gemcitabine through microbial enzymes (93). Throughout this review, we distinguish mechanisms supported by direct PDAC evidence from those inferred from other malignancies or systemic disease models, explicitly framing the latter as emerging and hypothesis-generating axes.

Emerging evidence positions the microbiome as a metabolic, immunologic, and stromal integrator of pancreatic carcinogenesis. High-resolution multi-omic datasets reveal that intratumoral microbial genes regulating nucleotide salvage and SCFA synthesis predict immunotherapy responsiveness and survival (31, 98). Likewise, microbiota-driven metabolic rewiring, particularly involving the tryptophan-kynurenine and bile-acid axes, defines the inflammatory and immunometabolic trajectories of PDAC progression (32). These insights expand the conventional view of dysbiosis beyond compositional shifts, highlighting functionally active microbial pathways that interface with oncogenic signaling and therapeutic resistance. At the same time, several of these functional links are more robustly established in colorectal and hepatobiliary cancers or metabolic disease than in PDAC, underscoring the need for dedicated pancreas-focused validation. In parallel, comparative work has exposed substantial cross-cohort and platform-dependent variability in microbial signatures, showing that many candidate “oncomicrobes” and risk scores lose discriminatory power when transferred across populations, especially when derived from 16S rRNA sequencing rather than shotgun metagenomics or multi-kingdom profiling. Consequently, future PDAC microbiome frameworks must integrate robust, harmonized pipelines (including shallow shotgun metagenomics, virome/mycobiome interrogation, and standardized analytical workflows) to move from cohort-specific associations toward reproducible, clinically actionable signatures (147, 161–163).

Beyond composition alone, an emerging temporal layer has been proposed to integrate the gut–pancreas axis with host clocks: peripheral circadian oscillators in the gut and pancreas coordinate immune tone, metabolic flux (e.g., bile-acid and tryptophan pathways), and barrier function, while diurnal microbiota rhythms reciprocally entrain clock gene programs in distal tissues (164–167). To date, these circadian–microbiome interactions have been demonstrated predominantly in metabolic and gastrointestinal disease models rather than in PDAC-specific cohorts. Disruption of this bidirectional circuit, by shift work, irregular feeding, or metabolic stress, induces dysbiosis, amplifies inflammatory signaling, and degrades antitumor immunity in experimental systems, raising the hypothesis that similar perturbations could contribute to pancreatic tumor promotion and therapy resistance (20, 165, 166). We therefore consider the circadian–microbiome–pancreas axis a conceptual, hypothesis-generating framework that requires targeted mechanistic and clinical studies in PDAC. Conceptually, microbe-host control of gene regulation extends to epigenetic remodeling: in virus-driven cancers, oncoproteins rewire DNA methylation and histone marks to sustain immune evasion and oncogenic transcriptional states, an instructive paradigm that underscores how exogenous biological agents can durably program host chromatin and suggests analogous, testable mechanisms for microbiome-linked PDAC biomarkers and interventions (111, 168).

Mechanistically, inflammation remains central. Microbiota-induced immune dysregulation, including T cell exhaustion and myeloid cell reprogramming, drives tumor progression and therapy failure (44). Certain bacteria, such as *Enterococcus faecalis* and *H. pylori*, have been shown to invade pancreatic tissue, exacerbating inflammation and mutagenesis (108). Bacterial colonization of the pancreatic TME is now recognized not as incidental but as functionally active. Intratumoral microbiota may directly contribute to chemoresistance through enzymatic drug degradation, particularly against gemcitabine, and modulate immunosurveillance. Microbiota-driven inflammatory circuits, particularly those involving LPS-TLR4 and downstream cytokine signaling, remain pivotal in sustaining immune evasion and tumor progression (52, 105, 112). Emerging research highlights that the microbiome also modulates therapeutic response. Strategies such as FMT, narrow-spectrum antibiotics, or probiotic regimens are being explored to improve immunotherapy outcomes or reverse drug resistance. These approaches are especially relevant in the era of precision oncology, where patient-specific microbial signatures could inform individualized treatments (14, 49, 71). Yet, recent low-biomass and contamination-aware re-analyses caution that some earlier associations between intratumoral bacteria and gemcitabine inactivation or ICI response may have overestimated microbial contribution, emphasizing the need to corroborate sequencing-based findings with culture, metabolomics, gnotobiotic models, and spatially resolved host–microbe mapping before translating them into routine clinical practice. Single-cell and spatial multi-omic studies that jointly profile fibroblasts, myeloid cells, lymphocytes, and microbial or microbial-derived signals are poised to refine which immune–stromal circuits are truly microbiome-dependent versus driven by tumor-intrinsic programs (169–171).

Looking forward, the microbiome stands as both a risk factor and therapeutic ally in PC, a duality that, if effectively decoded, may revolutionize prevention, early diagnosis, and individualized therapy in this highly lethal malignancy (20, 166). Harnessing multi-omic profiling to disentangle the complex host-microbiome-tumor crosstalk offers a promising avenue to identify mechanistic drivers and actionable biomarkers. Integrative analyses combining metagenomics, transcriptomics, metabolomics, and immunomics have already revealed microbial fingerprints with diagnostic and therapeutic relevance (21, 132). Prospective, longitudinal clinical trials are urgently needed to evaluate the efficacy and safety of microbiome-informed diagnostics and therapeutics in PC (111). In this context, enthusiasm for urine-based and serum-based biomarker panels must be tempered by evidence that diagnostic performance often attenuates in pre-diagnostic, screening-like settings compared with retrospective case–control cohorts, and that analytical variability across platforms can substantially affect sensitivity and specificity. Harmonized pre-analytical protocols, external quality assessment schemes, and regulatory-grade validation (including multi-center, multi-ethnic cohorts) will be required before microbiome-derived or microbiome-reflective biomarkers can be responsibly incorporated into PDAC surveillance algorithms for high-risk populations (75, 172).

The coming decade may well redefine PC management through the lens of microbial ecology. Integrative microbiome profiling will likely transform microbial ecosystems into actionable determinants of therapeutic precision, bridging the gap between microbial biology and individualized cancer management, and positioning microbiome modulation as a cornerstone of next-generation PC prevention and treatment (37, 167, 168). Key priorities will include (i) establishing international PDAC microbiome consortia with shared biobanking and harmonized sequencing pipelines; (ii) embedding microbiome endpoints into interventional trials of chemotherapy, radiotherapy, and immunotherapy; (iii) rationally designing microbiome-targeted interventions (live biotherapeutics, diet-based strategies, narrow-spectrum antibiotics, engineered phages, or FMT) with clear mechanistic hypotheses; and (iv) ensuring that data and analytical tools remain openly accessible to accelerate replication, meta-analysis, and translation. If these goals are met, the microbiome may transition from a descriptive biomarker of PDAC to a controllable axis of disease modification and therapeutic optimization.
